# Nonpharmacological Intervention Effects on Middle-Aged Women with Menopausal Symptoms: A Systematic Review and Meta-Analysis

**DOI:** 10.3390/healthcare13243206

**Published:** 2025-12-08

**Authors:** Ji-Hyun Kim, Hea-Jin Yu

**Affiliations:** College of Nursing, Sahmyook University, 815 Hwarang-ro, Nowon-gu, Seoul 01795, Republic of Korea

**Keywords:** middle-aged women, menopause, non-pharmacological

## Abstract

**Background:** Middle-aged women frequently experience diverse physical and psychological symptoms, including depression, anxiety, sleep disturbances, vasomotor symptoms, and reduced quality of life, during menopause. With increasing concerns regarding the side effects of hormone therapy, nonpharmacological interventions have emerged as safer alternatives for symptom management. **Purpose:** This systematic review and meta-analysis evaluated the effectiveness of nonpharmacological interventions for menopausal symptoms in middle-aged women. **Methods:** Thirty-two randomized controlled trials were selected from eight international and domestic databases. Interventions were categorized as nutritional or herbal, psychological, exercise-based, and complementary therapies. Risk of bias was assessed using the Cochrane risk of bias tool, and meta-analyses were performed using a random effects model. Results: Nonpharmacological interventions significantly reduced symptoms of depression (standardized mean difference (SMD) = −1.10), anxiety (−0.82), sleep disturbances (−0.90), menopausal symptoms (−1.18), and hot flashes (−0.34). Improvement in quality of life was observed, but it was not statistically significant (SMD = 1.40). Subgroup analyses revealed that nutritional and herbal interventions had the most consistent effects, and psychological and exercise-based interventions were particularly effective for improving sleep outcomes and emotional well-being. **Conclusions:** Nonpharmacological interventions effectively improve menopausal symptoms and quality of life, with tailored and multifaceted approaches showing the greatest impact. Nurse-led, community-based programs are key delivery platforms, and sustainable outcomes require standardized protocols, cultural responsiveness, and ongoing evaluation.

## 1. Introduction

Middle-aged women experience not only physical changes, such as aging and menopause, but also significant psychological shifts, including symptoms of depression and anxiety. This period of life is often marked by major transitional events, such as the evolving maternal role as children become independent, and is considered a critical turning point in a woman’s life [1].

Middle age was defined 40–65 years, consistent with reproductive biology and established frameworks such as STRAW+10 [2]. This range captures the menopausal transition and postmenopause, during which significant physiological changes occur and chronic disease risks increase [2]. Menopause typically occurs around 49–51 years, with onset as early as 40 [3,4], and the upper limit reflects late postmenopause when ovarian aging stabilizes.

Menopause is a natural biological process during which a woman’s ovaries gradually reduce hormone production, eventually leading to the permanent cessation of menstruation. These changes typically begin in the late 40s and progress over time [5]. In Korean women, the average onset of menopause has been reported to occur around the age of 51; the age of onset is clinically defined as the point at which a woman has experienced the absence of menstruation for 12 consecutive months [6].

The primary cause of menopause is a decline in ovarian function and a subsequent decrease in estrogen levels. As the ovaries become less active and produce less estrogen, the reproductive system, other vital organs such as the heart and brain, and bones are significantly affected [6]. Menopausal experiences among middle-aged women vary widely and can be influenced by multiple factors, including genetic predisposition, smoking habits, irregular lifestyle patterns, stress, medical treatments, and the presence of chronic illnesses such as hypertension and diabetes. These factors can accelerate the decline in ovarian function and exacerbate menopausal symptoms [7,8].

As women enter menopause, they undergo diverse physiological and psychological changes that are triggered by hormonal fluctuations. These transitions may serve as a significant life inflection point. Common physical symptoms include decreased skin elasticity, reduced libido, headaches, cardiovascular issues, hot flashes, sleep disturbances, and facial flushing [7,8]. Psychological symptoms frequently include increased irritability and depressive symptomatology, such as feelings of worthlessness, fatigue, lack of motivation, and diminished concentration [9,10,11].

Treatment strategies for menopausal symptoms can be broadly categorized into pharmacological and nonpharmacological interventions. Nonpharmacological interventions are increasingly emphasized because of their lower risk of adverse side effects and relatively safer profiles compared with hormone or drug therapy. As such, it is essential to explore nonpharmacological interventions as a priority when addressing the various symptoms experienced by menopausal women [6].

While menopausal hormone therapy (MHT) is highly effective for vasomotor symptom relief, the Women’s Health Initiative (WHI) trial demonstrated significant risks including increased breast cancer (HR: 1.26), venous thromboembolism (HR: 2.06), and cardiovascular disease [12,13]. Furthermore, MHT is contraindicated in women with personal history of breast cancer, thromboembolic disorders, or cardiovascular disease, thereby excluding a substantial proportion of symptomatic middle-aged women from this treatment option [14]. High discontinuation rates due to adverse effects such as breakthrough bleeding (40%), breast tenderness, weight gain, and mood disturbances further limit the real-world effectiveness of MHT [14]. Additionally, Lu et al. [4] reported that 77% of symptomatic Chinese women aged 40–60 years remained reluctant to receive MHT even after experiencing significant menopausal symptoms, primarily due to safety concerns and the perception of menopause as a natural process. Importantly, MHT addresses only hormonal deficiency without resolving underlying factors such as sleep disturbances, stress, autonomic nervous system imbalance, and lifestyle factors that contribute to menopausal symptom burden [15]. Therefore, non-pharmacological interventions—including lifestyle modifications, mind–body therapies, cognitive-behavioral therapy, and exercise—represent essential alternatives that offer safe, holistic approaches for women who cannot or prefer not to use MHT while addressing multiple symptom domains and promoting overall health during the menopausal transition [16,17].

Several types of nonpharmacological interventions have been examined. These include physical activity programs such as dance and movement therapies [18], art- or music-based interventions [19], counseling and relationship enhancement programs [20], and cognitive-based strategies such as cognitive training or stimulation interventions [21]. Despite the increasing number of studies in this domain, a comprehensive and systematic synthesis of the evidence related to the effectiveness of such interventions is required, particularly in terms of physical and psychological outcomes.

A systematic and integrated review of existing nonpharmacological interventions is warranted to objectively assess their effectiveness and provide a robust evidence base for selecting and implementing appropriate interventions for women experiencing menopausal symptoms. Through evidence synthesis, healthcare providers can develop well-informed strategies for clinical practice, supporting improved health outcomes for this population.

### Purpose of This Study

The purpose of this study was to systematically review and synthesize domestic and international research on nonpharmacological interventions designed for middle-aged women experiencing menopausal symptoms and to conduct a meta-analysis to estimate their overall effectiveness. Specifically, this study sought to identify the general characteristics of existing intervention studies, examine the types and components of nonpharmacological intervention programs currently being implemented for middle-aged women with menopausal symptoms, and evaluate the effectiveness of these programs in improving menopausal symptoms and related outcomes.

## 2. Materials and Methods

### 2.1. Study Design and Procedures

**Figure 1:**
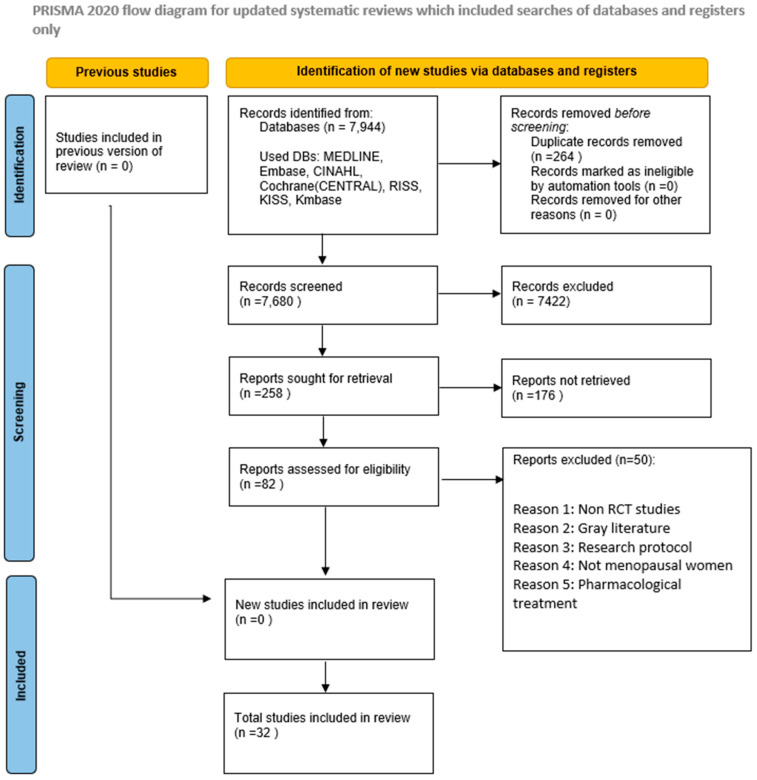
PRISMA Flowchart of the Included Studies.

This review was conducted following the Preferred Reporting Items for Systematic Reviews and Meta-Analyses (PRISMA) guidelines and the methodological standards detailed in the *Cochrane Handbook for Systematic Reviews of* Interventions [22]. A detailed summary of the study selection process is provided in the PRISMA flow diagram (Figure 1). Furthermore, the review protocol was prospectively registered on the International Platform of Registered Systematic Review and Meta-Analysis Protocols under the registration number INPLASY202570094.

The target population was middle-aged women experiencing menopausal symptoms.

- P (Population): Middle-aged women aged 40−65 years undergoing menopause
- I (Intervention): Nonpharmacological interventions
- C (Comparison): Individuals who did not receive the intervention or received a sham intervention
- O (Outcomes): Effects of the nonpharmacological intervention
- S-D (Study Design): Randomized controlled trials (RCTs)

#### 2.1.1. Inclusion Criteria

This review included studies that met the following eligibility criteria: (1) studies involving women aged 40−65 years in the perimenopausal stage, (2) studies that assessed the effectiveness of nonpharmacological interventions, (3) studies utilizing an RCT design, and (4) papers published in English or Korean.

#### 2.1.2. Search Strategy

To ensure the identification of relevant and high-quality studies, a comprehensive literature search was conducted across the following databases: Cumulative Index to Nursing and Allied Health Literature (CINAHL); Medline (PubMed); EMBASE; the Cochrane Central Register of Controlled Trials; and Korean databases such as the Korean Studies Information Service System, Research Information Sharing Service, and Korean Medical Database. The search terms included a wide range of relevant keywords, such as “climacteric,” “menopause,” “nonpharmacological intervention*,” “psychotherapy,” “cognitive behavioral therapy,” “cognitive therapy,” “behavioral therapy,” “relaxation therapy,” “meditation,” “imagery,” “directive counseling,” “psychological intervention,” “social support,” “therapy,” “counseling,” and “program.” Both free-text terms and controlled vocabulary (e.g., MeSH or Emtree) were used, and the terms were expanded or “exploded” as appropriate to enhance the comprehensiveness of the search. The full search dates (1 August 2023 to 12 August 2024) The queries for the search strategies are listed in Table S1.

#### 2.1.3. Study Selection and Data Extraction

Following the identification of relevant studies, any discrepancies were resolved through discussions between the two reviewers (JHK and HJY). A standardized approach was applied to systematically extract and organize the data concerning the study population as well as the details of the nonpharmacological interventions and control conditions. Trial characteristics were documented using a data extraction form based on the Cochrane Review guidelines.

#### 2.1.4. Risk of Bias

**Figure 2:**
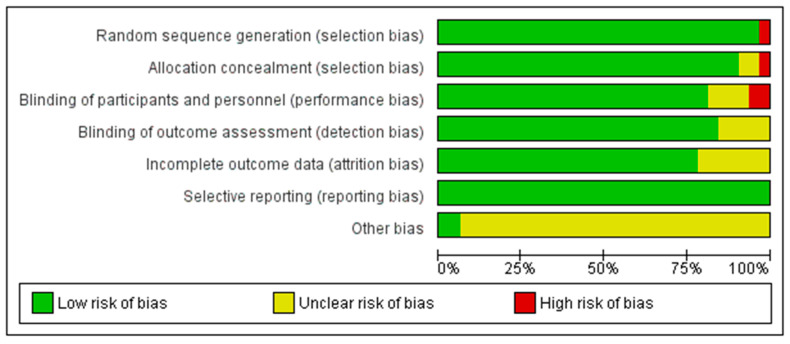

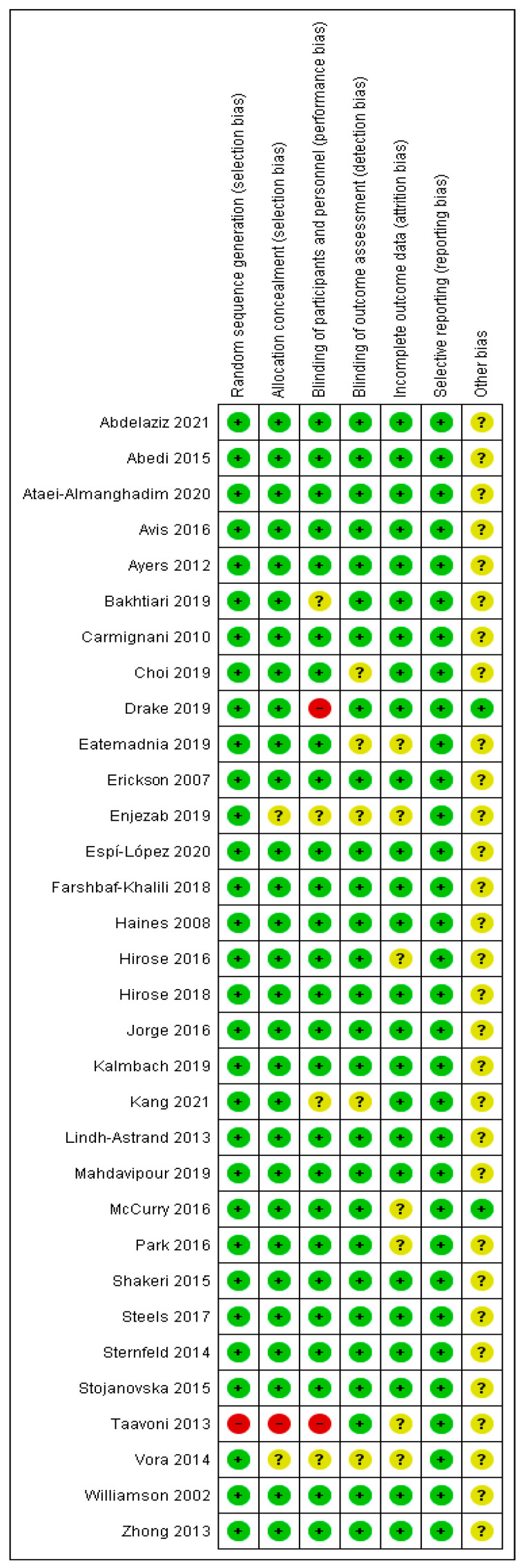
Risk of Bias of the Included Studies [25,26,27,28,29,30,31,32,33,34,35,36,37,38,39,40,41,42,43,44,45,46,47,48,49,50,51,52,53,54,55].

The methodological quality of the included studies was evaluated using the Cochrane risk of bias tool, which examines six domains: random sequence generation, allocation concealment, blinding of participants and personnel, blinding of outcome assessment, incomplete outcome data, and selective reporting [23]. Two authors (JHK and HJY) independently assessed the risk of bias, and any disagreements were resolved through discussion. A summary of the risk of bias assessment is provided in Figure 2.

For the data analysis, Review Manager version 5.3 was used to calculate the effect sizes, focusing on the impact of the psychological interventions on depressive symptoms and sleep quality. Standardized mean differences (SMDs) with 95% confidence intervals (CIs) and weighted mean differences were computed to estimate the overall effect sizes. A fixed effect model was employed, as the data were deemed sufficiently homogeneous, suggesting a consistent treatment effect across studies.

The Q-test and Higgins’ *I*^2^ statistics were used to assess statistical heterogeneity. Forest plots were generated to visually examine the effect sizes, CIs, and their overlap prior to conducting formal statistical tests. According to the criteria established by Higgins et al. [24], heterogeneity was classified as low (25%), moderate (50%), or high (75%). The Grading of Recommendations Assessment, Development and Evaluation (GRADE) assessment was also conducted and is reported in Table S2.

## 3. Results

### 3.1. Risk of Bias

All 32 RCTs were evaluated for risk of bias across six domains. In terms of random sequence generation, 31 studies (97%) were assessed as having a low risk of bias, whereas 1 study (3%) was judged to be at high risk. Allocation concealment was adequately described in 30 studies (94%), with 1 study (3%) rated as unclear and another (3%) as high risk due to insufficient methodological information.

Regarding the blinding of participants and personnel, 26 studies (81%) were considered to have a low risk, 4 studies (13%) had an unclear risk, and 2 studies (6%) were judged to be at high risk. For the blinding of outcome assessment, 27 studies (84%) demonstrated a low risk of bias, whereas 5 studies (16%) were rated as unclear. Incomplete outcome data were appropriately addressed in 25 studies (78%), whereas 7 studies (22%) did not provide sufficient information and were assessed as unclear. Finally, all 32 studies (100%) showed no evidence of selective reporting bias and were rated as low risk for this domain (Figure 2).

### 3.2. Characteristics of Included Studies

A total of 32 studies met the predefined inclusion criteria and were included in the present systematic literature review (Table 1). These studies were conducted across 13 countries, with the highest representation from Iran (*n* = 9), the United States (*n* = 7), South Korea (*n* = 3), Japan (*n* = 2), and the United Kingdom (*n* = 2). Other contributing countries included China, Portugal, Brazil, India, Spain, Egypt, Sweden, and Australia. This geographical diversity reflects the global interest in nonpharmacological interventions to manage menopausal symptoms. The South Korean studies included psychological (self-compassion program), exercise-based (Pilates), and herbal (Schisandra chinensis extract) interventions.

The international studies covered a broader range of intervention types, including nutritional or herbal therapies (e.g., soy isoflavone, maca, and red clover), exercise (e.g., yoga, walking, and aerobics), psychological approaches (e.g., cognitive behavioral therapy (CBT) and mindfulness-based cognitive therapy (MBCT)), mind–body practices (e.g., reflexology, massage, and aromatherapy), and alternative treatments such as acupuncture and Chinese herbal medicine.

The sample sizes of the included studies varied considerably, ranging from 20 to 248 participants. Most studies targeted women in the perimenopausal or postmenopausal stage, with the mean age of participants typically falling between 45 and 60 years. The nonpharmacological interventions identified across the 32 included studies were heterogeneous in nature and classified into four major categories based on their modality and therapeutic focus.

First, herbal and dietary supplementation interventions (*n* = 12) were the most frequent, including agents such as soy isoflavones, red clover, Schisandra chinensis, curcumin, maca, lavender, bitter orange, and Chinese herbal medicine. These interventions were primarily administered orally in capsule or extract form [25,26,28,30,31,32,33,34,35,55,57]

Second, mind–body and physical activity programs (*n* = 6) were also commonly employed. These included structured exercise interventions such as yoga, Pilates, reflexology, aromatherapy, and pedometer-guided walking [36,37,38,52,54,56]. The average intervention duration for these programs was approximately 11.3 weeks, with 27 sessions, lasting approximately 62.5 min per session.

Third, psychological and behavioral interventions (*n* = 7) focused on improving mental health and emotional regulation through structured programs such as CBT, MBCT, and self-compassion training [39,40,41,42,43,44,53]. The average intervention duration for these interventions was approximately 7.9 weeks, with 6.9 sessions, lasting approximately 48.3 min per session.

Finally, lifestyle and complementary therapies (*n* = 7), including aromatherapy massage, acupuncture, craniofacial massage, and applied relaxation techniques, were implemented in several trials. These interventions were typically delivered by trained therapists or alternative medicine practitioners in clinical or community-based settings [45,46,47,48,49,50,51]. The average intervention duration for lifestyle and complementary therapies was approximately 10.9 weeks, with 10.4 sessions, lasting approximately 35 min per session.

The intervention periods ranged from 4 weeks to 6 months, with session frequency and duration varying substantially across studies. The modes of delivery included both online [39,44] and offline sessions. Intervention providers ranged from clinical psychologists, nurses, and physicians to behavioral therapists and trained lay facilitators. The control conditions employed across the studies included placebo, usual care, waitlist control, no-treatment control, and active comparators. Most interventions were conducted in clinical settings; however, a few were implemented in community settings [26,42,46,50,52,53].

### 3.3. Outcomes and Measurement Instruments

The primary outcomes evaluated across the studies were broadly categorized as follows:

- Menopausal symptoms were commonly assessed using standardized and validated scales, including the *Menopause Rating Scale* (MRS), *Menopause-Specific Quality of Life Questionnaire* (MENQOL), *Greene Climacteric Scale* (GCS), and *Kupperman Index*. These instruments covered several somatic, psychological, urogenital, and vasomotor symptoms.
- Psychological symptoms, such as depression and anxiety, were measured using the *Beck Depression Inventory*, *Hospital Anxiety and Depression Scale*, and *State-Trait Anxiety Inventory*.
- Sleep-related outcomes were assessed using the *Insomnia Severity Index*; *Pittsburgh Sleep Quality Index*; and subjective sleep diaries reporting total sleep time, sleep onset latency, and sleep efficiency.
- Vasomotor symptoms, including hot flashes and night sweats, were typically evaluated through symptom diaries, frequency checklists, or visual analog scales.
- Overall quality of life (QOL) was assessed using general and menopause-specific instruments, such as the *Short Form-36*, *World Health Organization Quality of Life Scale*, and *Women’s Health Questionnaire*.

### 3.4. Meta-Analysis

Among the 32 RCTs included in this systematic review, only 5−6 studies per outcome were eligible for the meta-analysis. Studies were excluded from the meta-analysis if they did not report sufficient statistical data (e.g., means and standard deviations), used heterogeneous outcome measures, or lacked comparable intervention and control groups. These limitations restricted their inclusion despite methodological rigor. The follow-up periods ranged from immediate post-intervention assessments to long-term evaluations conducted up to six months after the completion of the intervention.

#### 3.4.1. Meta-Analysis Results for Menopausal Symptoms

**Figure 3:**
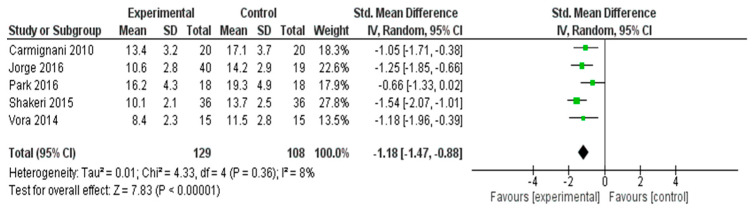
Meta-Analysis Results for Menopausal Symptoms [26,31,32,37,56].

A meta-analysis was conducted to evaluate the effectiveness of interventions in alleviating menopausal symptoms across five RCTs [26,31,32,37,56] (Figure 3). The pooled SMD, calculated using a random effects model, was −1.18 (95% CI: −1.47 to −0.88), indicating a statistically significant large effect favoring the experimental group (*Z* = 7.83, *p* < 0.00001).

Minimal heterogeneity was observed among the included studies (*I*^2^ = 8%, *τ*^2^ = 0.01, *χ*^2^ = 4.33, *df* = 4, *p* = 0.36), suggesting that the results were consistent across different study settings and populations. These findings demonstrate that the interventions had a substantial and reliable effect in reducing menopausal symptoms.

#### 3.4.2. Meta-Analysis Results for Hot Flash Symptoms

**Figure 4:**
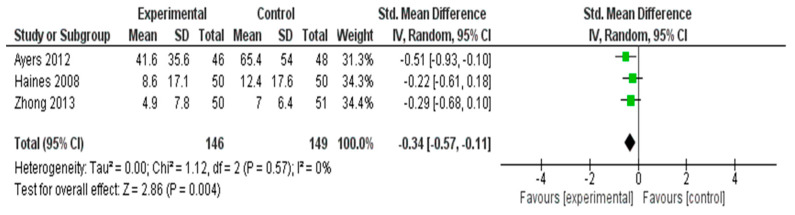
Meta-Analysis Results for Hot Flash Symptoms [35,40,55].

As hot flashes are one of the most frequently reported and distressing vasomotor symptoms among postmenopausal women, they were analyzed separately from other menopausal symptoms in this meta-analysis. Although hot flashes are commonly included as a subdomain within comprehensive menopausal symptom scales, such as the MRS or GCS, isolating this outcome allows for a more precise estimation of intervention effects on this high-priority symptom. A meta-analysis was conducted to evaluate the effectiveness of interventions in reducing hot flash symptoms across three RCTs [35,40,55] (Figure 4). The pooled SMD, calculated using a random effects model, was −0.34 (95% CI: −0.57 to −0.11), indicating a statistically significant small effect favoring the experimental group (*Z* = 2.86, *p* = 0.004).

No heterogeneity was observed among the included studies (*I*^2^ = 0%, *τ*^2^ = 0.00, *χ*^2^ = 1.12, *df* = 2, *p* = 0.57), suggesting consistency in intervention effects across trials. These results demonstrated that the interventions were modestly effective in alleviating hot flash symptoms in postmenopausal women.

#### 3.4.3. Meta-Analysis Results for Depressive Symptoms

**Figure 5:**
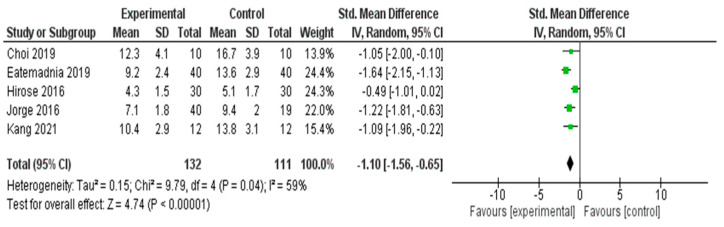
Meta-Analysis Results for Depressive Symptoms [29,37,52,53,57].

A meta-analysis was conducted to evaluate the effectiveness of the interventions on depressive symptoms across five RCTs [29,37,52,53,57] (Figure 5). The pooled SMD using a random effects model was −1.10 (95% CI: −1.56 to −0.65), indicating a statistically significant large effect favoring the experimental group (*Z* = 4.74, *p* < 0.00001).

Moderate heterogeneity was observed across the studies (*I*^2^ = 59%, *χ*^2^ = 9.79, *df* = 4, *p* = 0.04), suggesting some variability in effect sizes that may be due to the differences in intervention type, duration, or study populations. Nonetheless, the overall effect size remained robust and consistently favored the intervention group in reducing depressive symptoms.

#### 3.4.4. Meta-Analysis Results for Anxiety Symptoms

**Figure 6:**
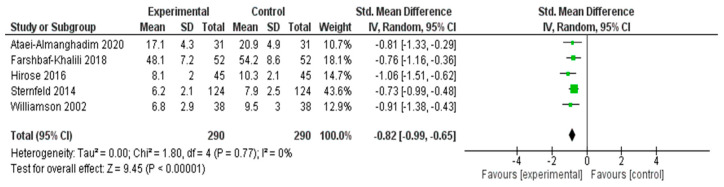
Meta-Analysis Results for Anxiety Symptoms [25,28,30,38,51].

A meta-analysis was performed to assess the effectiveness of nonpharmacological interventions in reducing anxiety symptoms across five RCTs [25,28,30,38,51] (Figure 6). The pooled SMD using a random effects model was −0.82 (95% CI: −0.99 to −0.65), indicating a statistically significant large effect favoring the experimental group (*Z* = 9.45, *p* < 0.00001).

No statistical heterogeneity was observed among the included studies (*I*^2^ = 0%, *χ*^2^ = 1.80, *df* = 4, *p* = 0.77), suggesting that the effect sizes were consistent across different interventions and populations. These results indicated that the interventions had a robust and beneficial impact on reducing anxiety symptoms in middle-aged women.

#### 3.4.5. Meta-Analysis Results for Sleep Outcomes

**Figure 7:**
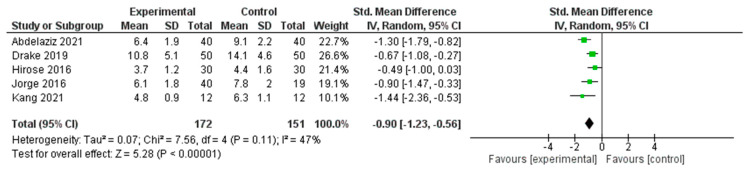
Meta-Analysis Results for Sleep Outcomes [29,37,39,41,52].

A meta-analysis was conducted to determine the effects of interventions on sleep quality across five RCTs [29,37,39,41,52] (Figure 7). The pooled SMD, calculated using a random effects model, was −0.90 (95% CI: −1.23 to −0.56), indicating a statistically significant large effect in favor of the experimental group (*Z* = 5.28, *p* < 0.00001).

Moderate heterogeneity was observed across the included studies (*I*^2^ = 47%, *τ*^2^ = 0.07, *χ*^2^ = 7.56, *df* = 4, *p* = 0.11), suggesting some variability in effect sizes; however, the overall direction of effect consistently favored the intervention group. These findings support the beneficial impact of nonpharmacological interventions on improving sleep outcomes in the studied populations.

#### 3.4.6. Meta-Analysis Results for Quality of Life

**Figure 8:**
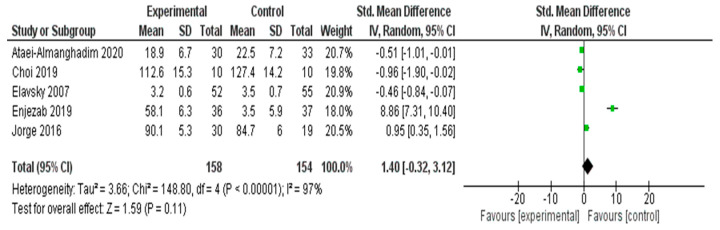
Meta-Analysis Results for Quality of Life [25,37,42,53,54].

A meta-analysis was conducted to assess the impact of interventions on QOL in five RCTs [25,37,42,53,54] (Figure 8). The pooled SMD, calculated using a random effects model, was 1.40 (95% CI: −0.32 to 3.12), suggesting a positive trend in favor of the experimental group. However, this effect was not statistically significant (*Z* = 1.59, *p* = 0.11). Substantial heterogeneity was detected across the included studies (*I*^2^ = 97%, *τ*^2^ = 3.66, *χ*^2^ = 148.80, *df* = 4, *p* < 0.00001), indicating significant variability in the intervention effects. This heterogeneity may be attributed to differences in the types of interventions, study populations, or QOL measurement tools. Given the wide confidence interval and high heterogeneity, caution should be exercised when interpreting the pooled effect size.

#### 3.4.7. Sensitivity Analysis for QoL

Prespecified leave-one-out sensitivity analyses demonstrated that the pooled effect for quality of life (QoL) was strongly influenced by a single outlying study. Removing Enjezab 2019 [57] reduced the pooled SMD from 1.40 (95% CI −0.32 to 3.12) to −0.22 (95% CI −0.94 to 0.50) and substantially lowered heterogeneity, although *I*^2^ remained high (≈85%). Omitting any other individual study did not materially change the effect estimates. Because each multi-arm RCT contributed only one effect size to the QoL analysis, robust variance estimation was not required.

The observed effects on QoL should be interpreted cautiously, as the direction and magnitude of the pooled estimate were largely driven by a single small trial with an extreme effect size. When this study was excluded, the pooled effect became small and non-significant. These findings are therefore hypothesis-generating rather than confirmatory, and well-powered RCTs are needed to clarify the true impact of non-pharmacological interventions on QoL.

### 3.5. Subgroup Analyses

#### 3.5.1. Menopausal Symptoms

The subgroup analysis of the five studies targeting overall menopausal symptoms revealed that nutritional and herbal interventions (*n* = 3) showed a substantial pooled effect (SMD ≈ −1.08 to −1.54), with consistent improvements in MRS scores. Exercise-based interventions (*n* = 2), including yoga and breathing practices, also demonstrated large effects (SMDs = −1.25 and −1.18). The overall pooled effect was strong and statistically significant (SMD = −1.18, 95% CI: −1.47 to −0.88), with very low heterogeneity (*I*^2^ = 8%). These findings suggested that both herbal and exercise-based interventions are highly effective in alleviating menopausal symptoms.

#### 3.5.2. Hot Flashes

The subgroup analysis of the three studies assessing nonpharmacological interventions for hot flashes revealed that herbal interventions (*n* = 2) showed small-to-moderate beneficial effects (SMDs = −0.22 and −0.29), with consistent results (*I*^2^ = 0%). The psychological intervention (group and self-help CBT; *n* = 1) demonstrated a larger individual effect (SMD = −0.51), but the subgroup-level estimation was not feasible. Overall, the pooled effect across all studies was statistically significant (SMD = −0.34, 95% CI: −0.57 to −0.11), suggesting that both herbal and psychological strategies can reduce vasomotor symptoms.

#### 3.5.3. Depressive Symptoms

Subgroup analyses were performed to compare the effectiveness of different types of nonpharmacological interventions for depressive symptoms. Nutritional or herbal interventions (*n* = 2) showed the largest pooled effect (SMD = −1.57), indicating a strong reduction in depression scores, followed by exercise-based and psychological interventions. Exercise-based interventions (*n* = 2) also demonstrated a substantial effect (SMD = −1.15). The psychological intervention group (*n* = 1) showed a significant individual effect (SMD = −1.05), but the subgroup estimation was limited by the analysis based on a single study. Among the three interventions, nutritional and herbal interventions appeared to be the most effective; however, interpretations should be made cautiously because of the small number of studies in each subgroup.

#### 3.5.4. Anxiety Symptoms

The subgroup analysis of the five studies assessing intervention effects on anxiety symptoms revealed that nutritional and herbal interventions (*n* = 3) demonstrated the strongest and most consistent effects (pooled SMD ≈ −0.88), with no observed heterogeneity (*I*^2^ = 0%). Interventions based on aerobic exercise (*n* = 1) and reflexology (*n* = 1) also showed beneficial effects individually (SMDs = −0.73 and −0.91, respectively); however, the subgroup comparison was limited due to the inclusion of only one study in each category. Overall, the findings suggested that nutritional and herbal approaches may offer the most robust benefits for alleviating anxiety symptoms in middle-aged women.

#### 3.5.5. Sleep Outcomes

The subgroup analysis of the five studies targeting sleep outcomes revealed that psychological interventions (*n* = 2), such as CBT for insomnia (CBT-I) and online CBT, demonstrated consistent and strong effects on sleep improvement (SMDs = −0.67 and −1.30). Exercise-based interventions (*n* = 2) also showed large effects (SMDs = −0.90 and −1.44). The nutritional intervention (*n* = 1) showed a moderate individual effect (SMD = −0.49). The overall pooled effect was statistically significant and substantial (SMD = −0.90, 95% CI: −1.23 to −0.56), with moderate heterogeneity (*I*^2^ = 47%). These findings suggested that exercise and psychological interventions may be highly effective in improving sleep in middle-aged women.

#### 3.5.6. Quality of Life

The subgroup analysis of five studies was conducted to evaluate the impact of different interventions on QOL. Psychological interventions (*n* = 2) showed the largest pooled effect, primarily driven by the study by Enjezab et al. (2019) [57] (SMD = 8.86), resulting in substantial heterogeneity (*I*^2^ = 97%). Exercise-based interventions (*n* = 2) demonstrated moderate effects (SMDs = 0.95 and −0.46), whereas the nutritional intervention (*n* = 1) had a small effect (SMD = −0.51). Owing to the extreme variance among effect sizes, particularly from one outlier study, the pooled effect was not statistically significant (SMD = 1.40, 95% CI: −0.32 to 3.12). These findings highlight the need for further well-controlled studies to clarify the impact of nonpharmacological interventions on the QOL of middle-aged women.

## 4. Discussion

### 4.1. Characteristics and Quality Assessment of Nonpharmacological Interventions for Menopausal Women

This study evaluated the effectiveness of nonpharmacological interventions for middle-aged women experiencing menopausal symptoms, thereby expanding the evidence base for such interventions and suggesting directions for future research. Nonpharmacological interventions were broadly categorized into four domains: nutritional or herbal, exercise-based, psychological, and complementary therapies (e.g., dietary supplementation, physical activity programs, psycho-behavioral interventions, and lifestyle or alternative approaches).

First, herbal and dietary supplementation interventions, including isoflavones, vitamin D, omega-3 fatty acids, and various herbal extracts, have been widely implemented. Several studies have reported the effectiveness of these interventions in alleviating vasomotor symptoms [26], improving QOL [27,46], and reducing depression and anxiety [58,59].

A meta-analysis by Oh et al. (2024) [60] involving perimenopausal women aged 38–85 years demonstrated notable results. Participants in the experimental group who consumed various herbal and dietary supplements (e.g., soy-derived isoflavones and red clover) experienced significant improvements in menopausal health status, such as reduced vasomotor symptoms and improved mood, sleep, and QoL, compared with the control group participants, as assessed by standardized tools such as the Kupperman Index. In addition, although the consistency and magnitude of the effects may vary depending on the specific components (e.g., isoflavones and red clover), several studies [61] have reported that such supplements yield beneficial changes in psychological symptoms, including anxiety and depression. Specifically, herbal agents such as licorice, valerian, sage, and ginseng have been repeatedly used to improve hot flashes, emotional symptoms, and sleep quality [62].

As such, compared with placebos, some herbal and dietary supplements have shown mild to moderate improvements in menopausal and psychological symptoms as well as QOL and appear to be generally safe in the short-term owing to their low incidence of side effects. However, the clinical efficacy remains moderate at best, and there is considerable heterogeneity in outcomes depending on the product, formulation, and study design. Thus, these supplements are recommended as complementary rather than primary treatments. Further investigations are needed to evaluate the additive effects of nutritional and dietary supplement interventions when combined with diet and exercise as well as their long-term safety profiles. Recent findings by Besong et al. (2024) [63] further support this interpretation. They reported that phytoestrogens effectively regulate hormonal fluctuations and provide moderate improvement in vasomotor symptoms while maintaining a high level of safety. These results reinforce the continued clinical relevance of herbal supplements such as isoflavones, red clover, licorice, sage, and ginseng. Notably, Besong et al. (2024) [63] emphasized that phytoestrogens pose a lower risk of adverse effects compared with hormone replacement therapy (HRT), underscoring their suitability as complementary therapeutic options. This aligns with the safety profile of herbal interventions identified in the present study and strengthens the rationale for their adjunctive use in menopausal symptom management.

Second, mind–body and physical activity programs, such as aerobic exercise, resistance training, yoga, and Pilates, have been extensively studied, and their effects have been reported to be significant in improving sleep quality [29,30,44], reducing depression and anxiety [46,64], alleviating vasomotor symptoms [34], and improving overall QOL and menopausal symptoms [38].

Thus, these interventions, besides reducing anxiety and depressive symptoms, may help stabilize the autonomic nervous system, leading to secondary benefits such as reduced cortisol levels, improved sleep, and enhanced immune function. Williamson et al. (2002) [51] reported that mental stability has a more substantial impact on QOL than physical symptoms. Therefore, multimodal interventions that incorporate education, emotional and social support, and nutritional management have been found to be more effective in alleviating menopausal symptoms than mind–body exercises alone [30,65]. However, physical activities such as yoga, aerobic exercise, and Pilates inherently pose structural limitations vis-à-vis study design, particularly in terms of blinding, which makes it crucial to ensure objectivity in randomization and outcome measurement. Moreover, the frequency and duration of interventions are critical variables in maximizing their effects. Hence, research designs that include both short-term interventions and long-term follow-up are essential. Third, CBT, mindfulness, and stress management interventions have demonstrated clinically significant effects on sleep disturbances and depressive, anxiety, and menopausal symptoms, representing the highest level of evidence among nonpharmacological treatments. The CBT programs have shown improvements in MENQOL and menopausal symptom indices [25,66].

In particular, CBT-I has demonstrated notable improvements in insomnia, depression, and QOL [41,44,67]. Sleep is not an isolated factor but serves as a nexus of cognitive, emotional, and physical health. Sleep disturbances in menopausal women are closely associated with cognitive decline and depression, making it essential to develop targeted interventions to address these issues. Numerous studies have found psychological and behavioral interventions to be effective in addressing psychological and emotional symptoms such as insomnia, depression, and anxiety. However, follow-up assessments over a 6–12-month period are necessary to evaluate the durability of these effects. Additionally, it is important to consider cultural and individual variations when designing interventions. For example, studies in Asian populations [42] have indicated that combining mind–body exercises with family and social support enhances intervention effectiveness. Furthermore, middle-aged women often face environmental challenges that hinder sustained motivation, and the presence of chronic conditions complicates the situation. Therefore, systematic approaches, including personalized interventions and trained health coaches, are necessary to ensure successful implementation.

Finally, alternative therapies such as lifestyle and health education, acupuncture, acupressure, music therapy, and aromatherapy have also been shown to alleviate vasomotor and menopausal symptoms, ultimately improving QOL. Notably, Avis et al. (2016) [43] found that acupuncture was effective in reducing the frequency of hot flashes and improving sleep and overall QOL. These findings suggested that the combined effects of psychological support and sleep-focused therapy may contribute to enhanced life satisfaction and well-being. Additionally, yoga interventions have demonstrated significant improvements in physical and psychological QOL [33]. Enjezab et al. (2019) [42] highlighted the significant role of non-pharmacological approaches in improving life satisfaction by encouraging menopausal women to take an active role in managing their own health. They also noted that differences in intervention effectiveness may depend on individual needs, group characteristics, and delivery format (e.g., online vs. in-person), underscoring the importance of accessibility considerations. In their evaluation of social support and physical activity programs for menopausal symptom relief, Enjezab et al. (2019) [42] emphasized that although physical activity itself is beneficial, interventions tend to be more effective when combined with peer support and structured group participation.

Similarly, Park et al. (2016) [31] assessed the effectiveness of health education and social support-based interventions in middle-aged Korean women and found that combining educational content with group-based support led to improvements in psychological and vasomotor symptoms as well as in QOL outcomes. Taken together, these findings suggested that the inclusion of social support significantly enhances the effectiveness of any intervention.

### 4.2. Effect Size of Nonpharmacological Interventions on Overall Menopausal Symptom Relief

Interventions such as CBT, yoga, mindfulness, structured exercise, and acupuncture were effective in improving key menopausal symptom scores (e.g., MENQOL and MRS). In particular, programs involving exercise and CBT conducted over 8–12 weeks or more have demonstrated significant improvements in overall QOL and specific menopausal symptom indices such as MENQOL [22,66].

In addition, various interventions such as meditation, dietary modification, and Pilates have been shown to alleviate menopausal symptoms and reduce depression and anxiety [19,40]. In alternative therapy groups, including those involving acupuncture and acupressure interventions, stable and substantial improvements in overall symptoms were reported [25,41] In particular, acupuncture, mindfulness, and structured exercise were consistently effective in alleviating hot flashes—a hallmark vasomotor symptom of menopause. Avis et al. (2016) [64] reported a significant reduction in the frequency of hot flashes in the acupuncture group compared with the placebo and waitlist control groups.

Additionally, regular physical activity significantly reduced the frequency and duration of vasomotor symptoms [32]. Beyond single interventions, a complex intervention combining group education and physical activity [28,58] resulted in reductions in vasomotor symptoms, including hot flashes.

Furthermore, these benefits were found to be maximized between 8 and 12 weeks after the intervention. Some studies [67] have demonstrated lasting effects that extended beyond six months. Various interventions such as yoga, meditation, acupuncture, and physical exercise have been effective in reducing hot flashes and overall menopausal symptoms, likely because of their role in modulating the autonomic nervous system dysregulation associated with estrogen fluctuations.

A Japanese RCT with 48 postmenopausal women receiving acupuncture revealed that a decline in estrogen led to heightened sympathetic nervous system activity and hypothalamic sensitivity related to thermoregulation [68]. This was reversed through acupuncture, restoring autonomic balance and alleviating vasomotor symptoms. Moreover, Newton et al. (2014) [69] conducted a study with perimenopausal women, who performed yoga and exercise twice weekly for 12 weeks. They found that, although yoga did not directly alter estrogen levels, mind–body practices based on breathing and meditation contributed to rebalancing sympathetic and parasympathetic nervous system activity, thereby reducing vasomotor symptoms.

### 4.3. Effect Size of Nonpharmacological Interventions on Depression and Anxiety in Menopausal Women

Among the nonpharmacological interventions, aerobic exercise and yoga yielded moderate to large improvements in anxiety and depression scores in middle-aged women [35,44]. A mindfulness-based group intervention by Carmignani et al. (2010) [26] also demonstrated significantly greater improvements in depression and anxiety measures in the experimental group than in the control group. This is plausible because aerobic exercise enhances neuroplasticity by increasing the secretion of neurotrophic and neurotransmitter factors (e.g., brain-derived neurotrophic factor, serotonin, and dopamine), thereby supporting the physiological remediation of depressive symptoms [54]. Furthermore, yoga and meditation are thought to stabilize the autonomic nervous system through regulated breathing and relaxation, resulting in physiological benefits such as lower cortisol levels, decreased heart rate, more stable blood pressure, and improved emotional stability [46]

Resilience enhancement programs [38] and mind–body relaxation-focused interventions [70] are particularly effective. Considering the strong psychosocial determinants of anxiety and depression, structured psychosocial support is warranted. Similarly, CBT-based programs have been shown to be highly effective against anxiety and related affective symptoms [56]. Moreover, regular exercise has consistently shown a positive effect on anxiety symptoms and emotional stability. Neurophysiologically, exercise promotes neuroplasticity in the hippocampus and prefrontal cortex, which promoted emotional stability and reduced depression. Furthermore, exercise-induced autonomic regulation, including heart rate regulation and breathing stabilization, contributes to stress response mitigation [32,35] involving postmenopausal women revealed that an intervention that combined diet and exercise regimes reduced inflammatory burden and dampened stress reactivity, thereby improving sleep quality and reducing anxiety.

Therefore, when considering nonpharmacological interventions for menopausal women, programs should intentionally integrate structured exercise and psychosocial support components to optimize mental health outcomes.

### 4.4. Effect Size of Nonpharmacological Interventions on Sleep Disorders in Menopausal Women

Nonpharmacological interventions, such as CBT, mindfulness, yoga, and acupuncture, have consistently been reported to be highly effective in improving sleep quality and alleviating insomnia [40,41,42]. Meta-analyses have provided strong evidence that CBT-I significantly improves sleep quality and reduces insomnia severity in menopausal women [71]; this is attributed to cognitive restructuring, which helps correct dysfunctional beliefs and behaviors regarding sleep, along with enhanced sleep hygiene practices. Systematic behavioral strategies, such as sleep restriction, stimulus control, and relaxation training, can stabilize the autonomic nervous system and effectively treat insomnia. Drake et al. (2019) [41] and Kalmbach et al. (2019) [72] reported that mindfulness practices and sleep hygiene education resulted in positive changes in sleep architecture and reduced daytime fatigue. In Japan, exercise, dietary modifications, and group-based interventions have been reported to be effective in improving sleep quality in middle-aged women [28,58]. These outcomes are attributed to the complex interaction of stabilizing the autonomic nervous system, improving vasomotor symptoms, and alleviating inflammation and musculoskeletal discomfort. As an alternative therapy, acupuncture has demonstrated beneficial effects on sleep quality [43], likely through the suppression of sympathetic overactivity and the enhancement of parasympathetic activation.

In addition to these behavioral and complementary approaches, recent pharmacological evidence has highlighted low-dose SSRIs as a potential non-hormonal option for addressing sleep disturbances associated with vasomotor symptoms. Riemma et al. [73] demonstrated that low-dose paroxetine significantly reduced hot flashes and improved sleep-related outcomes in both surgical and natural postmenopausal women. Although its effects were clinically meaningful, the presence of mild side effects and the limitations associated with medication-based approaches suggest that SSRIs may serve best as adjunctive rather than primary treatments when compared with the broader and more sustainable benefits provided by CBT-I, mindfulness, yoga, and other nonpharmacological interventions. Sleep disturbance is a key factor contributing to decreased QOL among menopausal women. Evidence suggests that these nonpharmacological sleep interventions enhance sleep quality and reduce sleep onset latency and the frequency of nocturnal awakenings. Furthermore, improved sleep has been associated with enhanced concentration, energy, and positive emotional functioning [42].

However, among these interventions, CBT-I, mindfulness [74], and yoga [75] were especially effective when delivered over longer durations—typically 6−12 weeks or more. In summary, sleep disturbances during menopause can be viewed as the outcome of a complex interplay between physiological, behavioral, and cognitive-emotional factors, requiring multifaceted interventions.

### 4.5. Effect Size of Nonpharmacological Interventions on the Quality of Life of Menopausal Women

Nonpharmacological interventions such as yoga, exercise, mindfulness, and CBT have been found to significantly improve QOL outcomes, assessed using tools such as the MENQOL, World Health Organization Quality of Life-BREF, and Short Form-36 Health Survey. These improvements are closely tied to the reduction in menopausal symptoms, suggesting that symptom alleviation directly enhances overall QOL.

As regular physical activity, dietary management, and sleep hygiene become integrated into daily routines, and as interest in and awareness of personal health grows, healthy behaviors are internalized, leading to a more sustainable QOL. Stojanovska et al. (2015) [34], who observed sustained health behavior reinforcement following aerobic exercise and dietary interventions, emphasized that establishing healthy habits is key to maintaining long-term improvements in QOL. Abdelaziz and Diab (2022) [39] and Lindh-Astrand et al. (2013) [48] further confirmed that both face-to-face and remote interventions are effective across various QOL domains (physical, psychological, and social). However, psychosocial factors such as social connections and support remain crucial for middle-aged women. Consequently, face-to-face, group-based interventions may be more effective than individual or virtual formats in fostering a sense of belonging and enhancing participants’ motivation to sustain their engagement.

Intervention providers in these studies included clinical psychologists, nurses, physicians, behavioral therapists, and trained laypersons. As nonpharmacological interventions require a multidimensional approach that addresses physiological, emotional, and psychosocial factors, nurses are considered particularly well-suited to serve as interventionists. Nurses are positioned to offer continuous support through education and personalized observation. Moreover, they possess the autonomy to implement noninvasive, health-promoting practices such as yoga, breathing exercises, meditation, sleep education, and nutritional guidance. Thus, nurses are expected to play a central role in leading nonpharmacological interventions.

Menopause represents a biological transition that significantly affects women’s long-term health. Enhancing self-care capabilities during this phase extends women’s healthy life expectancy as they approach older adulthood and produces broader societal benefits, as women often serve as primary caregivers within their families. Therefore, strengthening self-care skills among menopausal women can serve as a foundational strategy for fostering a healthier aging society.

Therefore, primary care systems should develop and implement preventive nursing interventions in collaboration with public and community health centers. Combined interventions, such as exercise paired with education or mindfulness, have been reported to be more effective than single-modality approaches [25]. Accordingly, there is a pressing need to develop standardized, integrative intervention protocols led by nurses that reflect the psychological, physical, and social contexts of menopausal women.

Group-based interventions such as exercise, yoga, and meditation alleviate physical symptoms, help mitigate social isolation, and strengthen mutual support systems. Avis et al. (2016) [45] found that peer interaction fosters psychological support, contributing to improved QOL. Therefore, rather than isolated individual interventions, it is essential to design tailored, integrative programs that reflect the sociopsychological shifts that accompany life transitions such as the empty nest syndrome. These programs should incorporate comprehensive health education on menopause, lifestyle modifications, cognitive and emotional regulation strategies, sleep hygiene education, and relaxation therapies, all delivered within a peer-supported, group-based framework. Furthermore, systematic monitoring and evaluation of the effectiveness of these nurse-led, nonpharmacological interventions are essential to ensure their impact and sustainability.

### 4.6. Limitations

This systematic review and meta-analysis underscores the utility of nonpharmacological interventions in managing menopausal symptoms among middle-aged women. However, several study limitations must be acknowledged. First, the inclusion of only peer-reviewed studies from international databases might have excluded gray literature, such as dissertations or nonindexed reports, potentially introducing publication bias. Second, the methodological quality of the included studies might have been affected by the inherent difficulty in blinding participants and researchers to the behavioral interventions. Although heterogeneity was partially addressed through subgroup analyses, variability in intervention types and durations might have influenced the interpretation of the results. Third, the predominance of short-term studies (typically 4–8 weeks) restricted insights into the long-term sustainability of the effects. Fourth, the lack of head-to-head comparisons of the type, frequency, and intensity of exercise modalities hindered the ability to identify the most effective interventions. Finally, the small number of culturally diverse studies restricted generalizability across populations and settings.

## 5. Conclusions

Nonpharmacological interventions for menopausal women, including CBT, mindfulness, physical activity, acupuncture, and nutritional therapy, have demonstrated statistically and clinically significant improvements in vasomotor symptoms, psychological well-being, sleep quality, and overall life satisfaction. These findings reinforce the value of integrative and holistic approaches in nursing practice.

Importantly, the magnitude and durability of intervention outcomes appear to be moderated by factors such as duration, frequency, cultural context, and the availability of social support. Therefore, interventions that are symptom-specific, multifaceted, and contextually tailored are likely to yield optimal results. Nurse-led, community-based programs should serve as pivotal platforms for delivering these interventions, bridging personalized care with public health outreach. Continued efforts to standardize intervention protocols, expand culturally responsive research, and embed evaluation mechanisms are critical for translating these findings into sustainable, real-world impacts.

## Figures and Tables

**Table 1:** Characteristics of the Included Studies.

Study (Author) Country	Sample Size (*N*)	Intervention (1) Mode of Therapy (2) Duration or Number of Sessions (3) Min/Session/or dosage (4) Provider (5) Mode of Delivery (6) Study Setting	Control	Follow-Up Times	Main Outcomes (Scale)
1. Farshbaf-Khalili et al. (2018) [28] Iran	156 (52 per group)	(1) Lavender and bitter orange capsules (2) 8 weeks (3) 500 mg/day (4) Nurse or researcher (5) Offline (6) Clinical	Placebo (starch capsules)	Baseline and 8 weeks	State-Trait Anxiety Inventory
2. Hirose et al. (2018) [30] Japan	96	(1) Soy lecithin (600 mg/day or 1200 mg/day) (2) 8 weeks (3) Daily (4) Physician (5) Offline (6) Clinical	Placebo	Baseline, 4 weeks, and 8 weeks	Profile of Mood States–brief form, Chalder Fatigue Scale, Brief Fatigue Inventory, Cardio-Ankle Vascular Index
3. Steels et al. (2017) [33] Australia	115 (Active *n* = 59, Placebo *n* = 56)	(1) Fenugreek extract (600 mg/day) (2) 12 weeks (3) Daily (4) Not specified (research team) (5) Offline (6) Not specified	Placebo	Baseline and 12 weeks	MENQOL, Hot flash frequency, Serum estradiol
4. Hirose et al. (2016) [29] Japan	90	(1) Isoflavone aglycone (12.5 mg/day or 25 mg/day) (2) 8 weeks (3) Daily (4) Physician (5) Offline (6) Clinical	Placebo	Baseline, 4 weeks, and 8 weeks	Hospital Anxiety and Depression Scale, Athens Insomnia Scale, Menopause Symptom Score
5. Choi et al. (2019) [53] South Korea	20 (10 in each group)	(1) Self-compassion program (2) 6 weeks (3) 60 min/session, weekly (4) Psychology professionals (5) Offline (6) Community	Waitlist	Baseline and post-intervention	Center for Epidemiologic Studies Depression Scale, Stress Response Inventory, MENQOL
6. Kang and Jeong (2021) [52] South Korea	36 (12 per group)	(1) Pilates exercise (2) 12 weeks (3) 50–55 min/session, 3 times/week (4) Exercise professionals (5) Offline (6) Community	Comparative and control groups	Baseline and post-intervention	Sleep quality, depression, fatigue (instruments not specified)
7. Enjezab et al. (2019) [42] Iran	73 (intervention *n* = 36, control *n* = 37)	(1) Mindfulness-based cognitive therapy (2) 8 weeks (3) 2 h/session, weekly (4) Certified professionals (5) Offline (6) Community	No intervention	Baseline, immediately post-intervention, 1 month post-intervention	MENQOL
8. Eatemadnia et al. (2019) [27] Iran	80 (40 per group)	(1) Hypericum perforatum (270–330 μg) (2) 8 weeks (3) Three times/day (4) Not specified (research team) (5) Offline (6) Clinical	Placebo	Baseline and 2, 4, 6, and 8 weeks	Kupperman Index, Hamilton Depression Rating Scale
9. Ataei-Almanghadim et al. (2020) Iran [25]	93 (curcumin, vitamin E, placebo)	(1) Curcumin 500 mg/day, vitamin E 200 IU/day (2) 8 weeks (3) Twice daily (4) Research team and physician (5) Offline (6) Clinical	Placebo	Baseline, 4 weeks, and 8 weeks	Hot flash checklist, Anxiety Scale, Female Sexual Function Index, Greene Climacteric Scale
10. Zhong et al. (2013) [35] China	108	(1) Chinese herbal therapy (Er-Xian decoction) (2) 12 weeks (3) Not stated (4) Chinese medicine practitioner (5) Offline (6) Clinical	Placebo	3 months post-intervention	Hot flash frequency or severity, MRS, MENQOL
11. Carmignani et al. (2010) Portugal [26]	60	(1) Dietary soy isoflavone (90 mg/day) (2) 16 weeks (3) Daily (4) Self-administered (5) Offline (6) Community	Placebo and low-dose hormone therapy	Post-treatment (16 weeks)	MRS (somatic, urogenital, and psychological)
12. Haines et al. (2008) USA [55]	100 (final *n* = 50 per group)	(1) Chinese herbal (Dang Gui Buxue Tang) (2) 6 months (3) Not stated (4) Chinese medicine practitioner (5) Offline (6) Clinical	Placebo	6 months	Hot flash frequency or severity, night sweats
13. Jorge et al. (2016) Brazil [37]	88	(1) Hatha yoga (2) 12 weeks (2 times/week) (3) 75 min/session (4) Certified yoga instructor (5) Offline (6) Clinical	Exercise and no-intervention control	Post 12-week intervention	MRS, BDI, World Health Organization Quality of Life Scale, hormone levels
14. Vora and Dangi (2014) India [56]	30	(1) Pranayama, Surya Namaskar (2) 4 weeks (3) Alternate days (4) Supervised by yoga therapists (5) Offline (6) Clinical	No intervention	Day 1 and Day 30	MRS, MENQOL
15. Erickson et al. (2007) USA [54]	164	(1) Walking/Yoga (2) 4 months (3) Not stated (4) Yoga instructors (5) Offline (6) Clinical	No-intervention control	Pre-post intervention	Mental health, menopause-related QOL
16. Park and Kim (2016) Republic of Korea [31]	36	(1) Schisandra chinensis extract (2) 6 weeks (3) Not stated (4) Clinical trial administration (5) Offline (6) Clinical	Placebo	12 weeks	Kupperman Index, MRS
17. Shakeri et al. (2015) [32] Iran	72	(1) Red clover (40 mg/day) (2) 12 weeks (3) Daily (4) Pharmacist or researcher (5) Offline (6) Clinical	Placebo	Pre-post intervention	MRS
18. Espí-López et al. (2020) Spain [47]	50	(1) Craniofacial massage (2) 4 weeks (3) Not explicitly stated (4) Physical therapist (5) Offline (6) Clinical	No intervention	Baseline, end of treatment, and 1 month follow-up	MRS, body image, mental health, QoL
19. Williamson et al. (2002) UK [51]	76	(1) Foot reflexology (2) 9 sessions over 19 weeks (3) Not specified (4) Qualified reflexologists (5) Offline	Nonspecific foot massage	19 weeks	WHQ: anxiety, depression; Visual Analogue Scale: hot flashes, night sweats
20. Avis et al. (2016) USA [45]	209	(1) Acupuncture (2) Up to 20 sessions over 6 months (3) Not specified (4) Certified acupuncturists (5) Offline (6) Clinical	Waitlist control (usual care)	6 and 12 months	Frequency of VMS (hot flashes), QoL (various scales)
21. Ayers et al. (2012) UK [40]	140	(1) Group and self-help CBT (2) 6-week and 26-week follow-up (3) Not specified (4) Clinical psychologists (5) Offline (6) Clinical	No treatment control	6 and 26 weeks	Hot flashes or night sweats problem rating, mood (WHQ), QoL (SF-36)
22. Abdelaziz and Diab (2022) Egypt [39]	80	(1) Internet-based CBT (2) 6 weekly modules (3) Not applicable (4) Self-administered online (5) Online (6) Clinical	Usual care	6 weeks	PSQI, ISI, sleep diary
23. Lindh-Astrand et al. (2013) Sweden [48]	60	(1) Applied relaxation (2) 12-week group therapy (3) 1 session/week (4) Trained therapists (5) Offline (6) Clinical	Untreated control	12-week and 3-month follow-up	Hot flash frequency, WHQ, salivary cortisol
24. Abedi et al. (2015) Iran [36]	106	(1) Pedometer walking (2) 12 weeks (3) Gradual step increase (4) Self-managed (5) Offline (6) Clinical	Usual care	4, 8, and 12 weeks	General Health Questionnaire-28, BDI
25. Sternfeld et al. (2014) USA [38]	248	(1) Aerobic exercise (2) 12 weeks, 3 times/week (3) Moderate intensity (4) Trainers (5) Offline (6) Clinical	Usual activity	6 and 12 weeks	VMS (daily diary), ISI, PSQI, Patient Health Questionnaire-8, Generalized Anxiety Disorder-7
26. Bakhtiari et al. (2019) Iran [46]	62	(1) Lavender aromatherapy inhalation (2) 20 min/night, 4 weeks (3) 20 min/session (4) Self-inhaled (5) Offline (6) Community	Placebo (distilled water)	4 weeks	MENQOL: vasomotor, physical, psychological, and sexual domains
27. Taavoni et al. (2013) Iran [50]	87 (final)	(1) Aromatherapy massage (2) 4 weeks, 2 times/week (3) 30 min/session (4) Trained nurses (5) Offline (6) Community	Massage without aroma or no treatment	4 weeks	MRS: psychological symptoms
28. Mahdavipour et al. (2019) Iran [49]	90	(1) Foot reflexology (2) 6 weeks, 2 times/week (3) 30 min/session (15 each foot) (4) Reflexologist (5) Offline (6) Clinical	Routine care	6-week and 2-month follow-up	BDI
29. Drake et al. (2019) USA [41]	150	(1) CBTI, SRT (2) CBTI: 6 weeks, SRT: 2 weeks (3) CBTI: ~30 min/session, SRT varied (4) Registered nurse sleep specialists (5) Offline (6) Clinical	SHE	Post-treatment and 6 months	ISI, sleep diary (total sleep time, SE%, wake after sleep onset, etc.)
30. Kalmbach et al. (2019) USA [43]	117	(1) CBTI, SRT (2) CBTI: 6 weeks, SRT: 2 weeks (3) CBTI weekly, SRT 2 face-to-face and 3 calls (4) Behavioral sleep medicine registered nurse (5) Offline and telephone follow-up (6) Clinical	SHE	Post-treatment and 6 months	BDI-II, Dysfunctional Beliefs and Attitudes about Sleep, Pre-Sleep Arousal Scale (cognitive and somatic), Emotion Regulation Rating Index, Penn State Worry Questionnaire
31. McCurry et al. (2016) USA [44]	106	(1) Telephone-based CBT for insomnia (2) 6 sessions over 8 weeks (3) 20–30 min/session (4) Trained coaches (MSW/PhD) (5) Online (6) Clinical	Menopause Education Control	8 and 24 weeks	ISI, PSQI, sleep diary, Hot Flash Interference Scale
32. Stojanovska et al. (2015) Australia [34]	29	(1) Oral Maca (Lepidium meyenii) capsules (2) 6 weeks (cross-over) (3) 3.3 g/day (7 capsules/day) (4) Self-administered (5) Offline (6) Clinical	Placebo (rice flour in identical capsules)	Baseline, 6 weeks, and 12 weeks	Greene Climacteric Scale, SF-36 v2, WHQ

Abbreviations: BDI: Beck Depression Inventory; BDI-II: Beck Depression Inventory-II; CBT: cognitive behavioral therapy; ISI: Insomnia Severity Index; MENQOL: Menopause-Specific Quality of Life Questionnaire; MRS: Menopause Rating Scale; PSQI: Pittsburgh Sleep Quality Index; QoL: quality of life; SF-36: 36-Item Short Form Survey; SHE: Sleep Hygiene Education; SRT: Sleep Restriction Therapy; VMS: vasomotor symptoms; WHQ: Women’s Health Questionnaire.

## Data Availability

The dataset supporting the conclusions is available from the corresponding author on reasonable request. The data are not publicly available due to privacy concerns.

## Supplementary Materials

The following supporting information can be downloaded at: https://www.mdpi.com/article/10.3390/healthcare13243206/s1, Table S1: Search Strategies; Table S2: GRADE Findings.

## Abbreviations

The following abbreviations are used in this manuscript: BDI: Beck Depression Inventory; BDI-II: Beck Depression Inventory-II; CBT: cognitive behavioral therapy; ISI: Insomnia Severity Index; MENQOL: Menopause-Specific Quality of Life Questionnaire; MRS: Menopause Rating Scale; PSQI: Pittsburgh Sleep Quality Index; QoL: quality of life; SF-36: 36-Item Short Form Survey; SHE: Sleep Hygiene Education; SRT: Sleep Restriction Therapy; VMS: vasomotor symptoms; WHQ: Women’s Health Questionnaire.
