# Supplementary material for: Nonpharmacological Intervention Effects on Middle-Aged Women with Menopausal Symptoms: A Systematic Review and Meta-Analysis
Source: Healthcare (Basel). 2025 Dec 8;13(24):3206. doi: 10.3390/healthcare13243206 (PMC12732411; doi:10.3390/healthcare13243206)
Supplement: Supplementary file 1 [file healthcare-13-03206-s001.zip › Table S1.pdf]

Supplement Table S1. Search Strategies

Database	Search Query (English)
**PubMed (MEDLINE)**	*("Menopause"[Mesh] OR menopause OR climacteric OR perimenopause OR postmenopause) AND ("Therapy, Nonpharmacologic"[Mesh] OR nonpharmacological OR "non-pharmacologic" OR psychological OR "cognitive behavioral therapy" OR CBT OR mindfulness OR exercise OR yoga OR pilates OR acupuncture OR herbal OR supplement OR complementary) AND ("Randomized Controlled Trial"[Publication Type] OR randomized OR randomised OR RCT)**
**CINAHL (EBSCO)**	*(MH "Menopause+" OR menopause OR climacteric OR perimenopause) AND (MH "Complementary Therapies+" OR nonpharmacological OR psychological OR CBT OR mindfulness OR exercise OR yoga OR pilates OR acupuncture OR herbal OR supplement OR aromatherapy OR reflexology) AND (MH "Randomized Controlled Trials" OR randomized OR randomised OR RCT)**
**EMBASE**	*('menopause'/exp OR menopause OR climacteric) AND ('non pharmacological therapy'/exp OR 'complementary therapy'/exp OR nonpharmacological OR psychological OR 'cognitive behavioral therapy'/exp OR CBT OR mindfulness OR exercise OR yoga OR pilates OR acupuncture OR 'herbal medicine'/exp OR supplement) AND ('randomized controlled trial'/exp OR randomized OR randomised OR RCT)**
**Cochrane CENTRAL**	*(menopause OR climacteric OR perimenopause) AND (nonpharmacological OR psychological OR CBT OR mindfulness OR exercise OR yoga OR pilates OR*

Database	Search Query (English)
	*acupuncture OR herbal OR supplement OR complementary) IN Trials**
**KISS (한국학술정보)**	**(갱년기 OR 폐경 OR 중년 여성) AND (비약물 OR 인지행동치료 OR 심리치료 OR 운동 OR 요가 OR 필라테스 OR 한약 OR 보완대체요법) AND (무작위 OR RCT)**
**RISS**	**(갱년기 OR 폐경) AND (비약물 OR 심리 OR 운동 OR 한방 OR 대체요법) AND (무작위 OR RCT OR 임상시험)**
**KMbase (한국의학데이터베이스)**	**(폐경 OR 갱년기) AND (비약물 OR 인지행동치료 OR 심리 OR 운동 OR 요가 OR 침 OR 한약 OR 허브 치료) AND (임상시험 OR 무작위배정)**
