# Supplementary material for: Nonpharmacological Intervention Effects on Middle-Aged Women with Menopausal Symptoms: A Systematic Review and Meta-Analysis
Source: Healthcare (Basel). 2025 Dec 8;13(24):3206. doi: 10.3390/healthcare13243206 (PMC12732411; doi:10.3390/healthcare13243206)
Supplement: Supplementary file 1 [file healthcare-13-03206-s001.zip › Table S2.pdf]

Supplement Table S2

GRADE Findings

Outcome	No. of RCTs	Effect (SMD, 95% CI)	ROB	Inconsistency (I^2)	Imprecision	Publication Bias	GRADE Certainty
**Menopausal symptoms**	5	–1.18 (–1.47, –0.88)	Mostly low	**8% (not serious)**	Not serious	Undetected	**Moderate**
**Hot flashes**	3	–0.34 (–0.57, –0.11)	Mostly low	**0% (not serious)**	Borderline small effect	Possible	**Low**
**Depression**	5	–1.10 (–1.56, –0.65)	Mostly low	**59% (serious)**	Moderate	Possible	**Low**
**Anxiety**	5	–0.82 (–0.99, –0.65)	Mostly low	**0% (not serious)**	Not serious	Undetected	**Moderate**
**Sleep quality**	5	–0.90 (–1.23, –0.56)	Mostly low	**47% (serious)**	Not serious	Possible	**Low**
**Quality of life**	5	1.40 (–0.32, 3.12)	Mostly low	**97% (very serious)**	Very wide CI	Likely	**Very Low**
